# The Future of 3D Brain Cultures in Developmental Neurotoxicity Testing

**DOI:** 10.3389/ftox.2022.808620

**Published:** 2022-01-27

**Authors:** Helena T. Hogberg, Lena Smirnova

**Affiliations:** Center for Alternatives to Animal Testing (CAAT), Johns Hopkins Bloomberg School of Public Health, Baltimore, MD, United States

**Keywords:** 3D brain model, DNT, neurotoxicity, brain organoids, brain MPS

## Abstract

Human brain is undoubtedly the most complex organ in the body. Thus, it is difficult to develop adequate and at the same time human relevant test systems and models to cover the aspects of brain homeostasis and even more challenging to address brain development. Animal tests for Developmental Neurotoxicity (DNT) have been devised, but because of complex underlying mechanisms of neural development, and interspecies differences, there are many limitations of animal-based approaches. The high costs, high number of animals used per test and technical difficulties of these tests are prohibitive for routine DNT chemical screening. Therefore, many potential DNT chemicals remain unidentified. New approach methodologies (NAMs) are needed to change this. Experts in the field have recommended the use of a battery of human *in vitro* tests to be used for the initial prioritization of high-risk environmental chemicals for DNT testing. Microphysiological systems (MPS) of the brain mimic the *in vivo* counterpart in terms of cellular composition, recapitulation of regional architecture and functionality. These systems amendable to use in a DNT test battery with promising features such as (i) complexity, (ii) closer recapitulation of *in vivo* response and (iii) possibility to multiplex many assays in one test system, which can increase throughput and predictivity for human health. The resent progress in 3D brain MPS research, advantages, limitations and future perspectives are discussed in this review.

## Introduction

### Overview of Brain Microphysiological Systems

**FIGURE 1:**
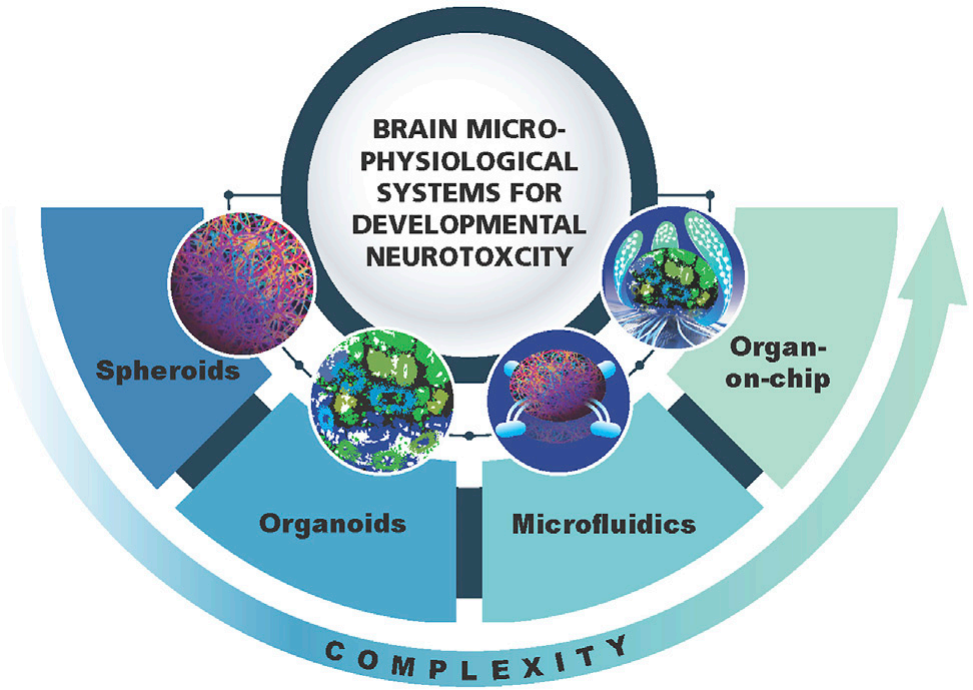
Schematic representation of 3D brain models as a component of brain microphysiological systems (brain MPS). Different levels of complexity (spheroids, organoids, microfluidics, brain-on-chip) are shown.

Microphysiological systems (MPS) have emerged over the last years and are representing new, more physiologically relevant cell cultures recapitulating organ architecture and functionality (Marx et al., 2020). The MPS can have different levels of complexity going from simpler spheroids to organoids, microfluidics and organs-on-chip (Figure 1). The term MPS in this review refers to any of these models with the focus on 3D brain models or brain organoids (Lancaster and Knoblich, 2014; Pasca et al., 2015; Di Lullo and Kriegstein, 2017; Koo et al., 2019; Shou et al., 2020; Sun et al., 2021). The main features of brain organoid cultures are representation of the *in vivo* brain in terms of cellular composition, recapitulation of regional architecture (e.g., cortical layers) and functionality (e.g., active synapses, electrical activity and myelination). The brain MPS are now broadly used to study neurological disorders, brain development and aging (Di Lullo and Kriegstein, 2017; Koo et al., 2019; Shou et al., 2020; Sun et al., 2021). Although, as all *in vitro* models, the MPS also have limitations. Researchers are working on advancing the MPS (second generation MPS) (Marx, 2020; Marx et al., 2020). Recent advances in 3D brain models are (i) combining organoids from different regions of the brain (Kim et al., 2019), recapitulating the connection between different types of neurons or neurons and muscles - assembloids (Andersen et al., 2020; Miura et al., 2020), (ii) modeling of chorion plexus (Pellegrini et al., 2020a) and organoids secreting cerebral spinal fluid (Pellegrini et al., 2020b) (iii) incorporation of immunocompetent cells—microglia (Abreu et al., 2018; Ormel et al., 2018; Bodnar et al., 2021) (iv) vascularization of brain organoids (Cakir et al., 2019; Ham et al., 2020) and models of brain organoids with blood brain barrier (BBB) (Bergmann et al., 2018; Nzou et al., 2020). However, in neurotoxicology, the reproducibility of the system is of highest importance, and therefore the developers are aiming for simplicity as far as possible but complex enough to recapitulate human-relevant cellular processes and functionality. It is clear that simple monolayer cultures have limitations and are far away from representing the human brain in terms of architecture and functionality, but they can still be very useful when combined in a battery of tests. The same applies for brain MPS. The main question to be asked here: how to find the balance between the complexity and simplicity needed to have robust, reproducible systems that can be applied for chemical screening in a higher throughput manner.

### Why are New More Advanced Cell Models Needed in (Developmental) Neurotoxicology?

Developmental Neurotoxicity (DNT) is an issue not adequately covered by existing testing strategies. Current DNT testing for risk assessment purposes is entirely animal-based and is not a standard requirement, not because of a lack of interest in the hazard but a lack of adequate testing opportunities (Smirnova et al., 2014). An important restriction for routine DNT assessment of drugs and chemicals are the high costs of the current regulatory test strategy according to the DNT guidelines (OECD TG 426 and US EPA 712-C-98-239) (EPA, 1998; OECD, 2007). But there are also scientific concerns regarding the relevance of these studies for human health effects. As current guidelines often do not provide sufficient information to facilitate regulatory decision-making, new approach methodologies (NAMs) to assess DNT are considered (EPA, 2020; Masjosthusmann et al., 2020). Experts in the field have recommended the use of a battery of *in vitro* tests covering the cellular key events of neural development to be used for the initial prioritization of high-risk environmental chemicals for DNT testing (Bal-Price et al., 2018a; Bal-Price et al., 2018b). These tests, are mostly based on traditional monolayer cell cultures, have been developed in different laboratories and measure different endpoints of DNT. This means the important step of harmonization and combination of the tests is necessary. Moreover, the important interactions between different cell types and key events during development are often missing. For this reason, fewer more complex unified MPS covering most key neurodevelopmental events would streamline this process of DNT testing. Some of these key events e.g., myelination and synapse formation might be better modeled in 3D. Myelination is a complex process which is hard to achieve *in vitro*, especially in 2D cultures. Few brain and spinal cord organoids have been developed that show differentiation and maturation of oligodendrocytes with the formation of the myelin sheath (Pamies et al., 2017; Madhavan et al., 2018; Chesnut et al., 2021; James et al., 2021; Shaker et al., 2021). Active synapses can be formed in both 2D and 3D cultures, where synaptogenesis can be assessed with high content imaging (Harrill et al., 2011; Verstraelen et al., 2018) or multi-electrode arrays (Brown et al., 2016). However, synaptogenesis in 3D can be modeled in more physiologically relevant cellular organization (network formation, pattering and layering of different brain regions described in organoids, which better reflects *in vivo* tissue complexity). One might suggest that there are certain advantages in studying synaptogenesis in 3D, although the analysis becomes more complex (see below in optimization of assays).

Although 3D models have been developed, very few compounds have been tested in these systems and there is currently no well-developed DNT test available using these human models (Bal-Price et al., 2018a). Thus, it is the most pressing to develop harmonized, human-relevant and relatively simple-to-use, transferable MPS for DNT.

## Challenges and Opportunities for 3D Brain Models

Even though 3D brain models have great potential to enhance the DNT assessment by more closely mimicking the *in vivo* situation, there are currently several limitations and challenges to address (Table 1).

**TABLE 1:** Summary of current challenges and future directions of 3D brain models.

Current challenges	Ongoing efforts and future directions
•Standardization and reproducibility	•Incorporation of immune system
•Cost and complexity	•Barrier models and vascularization
•Throughput	•Cellular composition optimization
•Optimization of assays	•Increased use of patient derived iPSCs
•Long differentiation and maturation	•Single cell measurements in MPS
•Not physiological ratio of the main cells	•Organ-organ interactions

### Standardization and Reproducibility

The major challenge to move 3D brain models towards regulatory acceptance for DNT testing is standardization and reproducibility. The more complex the system is the more rigorous quality control steps must be taken to ensure the system’s validity and predictivity of the outcomes. Although the reproducibility of brain organoids has been improving over the last years (Velasco et al., 2019; Yoon et al., 2019), especially through harmonization of the existing protocols, and commercially available kits for differentiation (e.g., StemCell Technology kit for cortical organoids), they are still heterogeneous cultures due to the nature of the system development: diversity of protocols, increased variability because of different donors of iPSC etc. The recent updated guidance document on Good Cell and Tissue Culture Practice 2.0 (GCCP 2.0) aims to provide guidance in assuring the reproducibility of *in vitro* systems, including the complex ones such as iPSC-derived models and MPS (Pamies et al., 2022). When it comes to different donors, the reproducibility between the cell lines is the key, as it has been shown that already on iPSC level, there is a high level of donor-to-donor, or even clone-to-clone variability (Volpato and Webber, 2020). The scientists are refining the protocols to improve the physiological relevance and to generate organoids from different parts of the brain (e.g., cerebellar organoids by Qian et al., 2016; Quadrato et al., 2017; Silva et al., 2020). The 3D structure complicates the reproducibility further as most techniques, e.g., gyratory shaking, scaffolding, hanging drop techniques, and spontaneous aggregating, allow cells within the organoids to migrate to its positions and initiate the self-organized cell-cell interactions. Since these models lack the complete chemotaxis present during development *in vivo* the variability between the organoid cell-architecture can be high. Several groups are using microfluidic chip-platforms to explore how the gradients of different substances such as growth factors and chemokines can control the migration and differentiation of cells (Kilic et al., 2016; Cho et al., 2021). The use of bioprinting methods are other ways to force cells into specific positions and shapes (Han and Hsu, 2017; Khan et al., 2021; Roversi et al., 2021), however, this can lead to artifacts as the cell’s natural potential might be restrained. All revisions of existing protocols for differentiation and cell culturing need to go through new standardization processes as even small changes can introduce new variables.

### Cost and Complexity

Another limitation when considering these systems for toxicology are still relatively high costs and complex protocols, especially for organ-on-chip systems. The protocols for human iPSCs-derived brain organoids are naturally long as those systems recapitulate human brain development, and the differentiation and maturation of different lineages *in vivo* is a long process stretching over the whole embryonic, fetal and first years of postnatal development (Rodier, 1980; Rice and Barone, 2000). Since most protocols currently are using iPSC cells, adequately trained cellular biologists are needed to complete the task. Growth factors and supplements are costly but are required for models which sometimes are kept in culture for up to a year (Lancaster and Knoblich, 2014; Madhavan et al., 2018; Trujillo et al., 2019; Giandomenico et al., 2021). For pharmaceutical and industrial companies, the complex MPS models might be more feasible on contract research organizations (CROs) bases than setting it up in house, as many MPS are still in a developing stage. By outsourcing the research and development activities to a third party, the companies can stay competitive and flexible in terms of novel techniques and profit (Clearwater International, 2021). Most academic labs developing new cell models and assays do not have the required quality management or expertise to provide the expected level of services. Moreover, for an assay to be useful in a regulatory context, the transferability to another laboratory is generally needed as part of the validation process. This can be challenging if the cell model is too complex and/or need specific laboratory equipment. The National Center for Advancing Translational Sciences (NCATS), National Institute of Health (NIH) awarded two Tissue Chip Testing Centers (https://ncats.nih.gov/tissuechip/projects/centers/2018) with the aim to independently reproduce previous published MPS data to assess their robustness, portability of the technology, develop best practices, and provide input for further improvement (Low and Tagle, 2017). Even though these new technologies need performance accreditation, the classical validation process will have to be adjusted to a fit-for-purpose validation (discussed in NAS, 2017; Marx et al., 2020).

### Throughput

One of the advantages with the *in vitro* approach is the increased throughput for testing chemicals. Many of the regulatory programs such as ToxCast (https://www.epa.gov/chemical-research/toxcast-chemicals) and Tox21 (https://tox21.gov/) are using robotic assays that can screen thousands of chemicals in a very short time. However, the cell systems used with these assays are often simple. Due to the complex nature of 3D cultures and MPS the throughput is still a limitation. One way to overcome this, is to develop microfluidic and automatic handling machines adapted for the organoids. Although brain-on-chip and microfluidic systems of neural cultures exist, they are limitedly utilized for 3D brain models (reviewed in Miccoli et al., 2018; Osaki et al., 2018). The use of more automatic culturing techniques will likely increase the reproducibility and standardization as well.

### Optimization of Assays

Most of the *in vitro* assays have been developed for monolayer cultures and therefore need to be adapted for the 3D systems. In general, before applying any assay developed for monolayer cultures, an extensive quality control of the intended application in 3D needs to be established. Two of them will be discussed as examples. Immunohistochemistry is labor intense and technically challenging in the 3D cultures. Some limiting factors include issues with antibody penetration and laser power of a confocal microscope which makes it difficult to image through the entire depth of an organoid. To overcome this, many groups are making cryosections of the organoids (Pamies et al., 2020), however, with the risk to damage the neurites or cell-cell interactions. Another option is tissue clearing processing which is required to obtain high quality images (Lallemant et al., 2020). However, this process is labor intensive, and time consuming. The enhanced solution could be generation of reporter lines, which may allow to follow different cell linages during development and after toxicant treatment. Imaging the reporter lines in combination with tissue clearing can overcome some of the laborious and expensive antibody staining and cryosectioning methods. Such approach is demanding during the development stage but easy to adapt, when ready to use, it can also increase the throughput and enable high content imaging for several processes in parallel such as neuronal differentiation, synaptogenesis, gliosis/gliagenesis, oligodendrogenesis and myelination, neurite formation and outgrowth. However, to improve the reproducibility and standardization, the introduction of reporter genes should be harmonized between 3D models (Fischer et al., 2019).

Multi-electrode array (MEA) is the most common assay used to assess neuronal functionality by measuring the electrical activity in the test system. Traditional MEA plates were designed for monolayer cultures and are broadly used in neuroscience (Halliwell et al., 2021; Taga et al., 2021; Tukker and Westerink, 2021). Furthermore, MEA has been applied to assess neurotoxicity and DNT in high throughput manner (Strickland et al., 2018; Shafer et al., 2019). However, these plates, have major limitations for 3D models. The density of electrodes is low, and the recording occurs only from the area, where the organoid is touching the electrode. It is difficult to get reproducible results, as it is technically hard to plate the organoids exactly the same way from well-to-well and plate-to-plate. One solution is to design an organoid EEG, a multielectrode shell, covering the whole surface of an organoid (Cools et al., 2018). High density multielectrode array might also offer a solution of more robust and reproducible recording in 3D (Sharf et al., 2021). Use of optogenetics to manipulate neural activity within brain organoids is also growing. The more advanced step would be to grow the organoids around the electrodes, so the recording can occur not only from the surface but also from the inside of the organoid. Advances of electrophysiology of brain organoids are further discussed in (Passaro and Stice, 2020).

### Translation to *in vivo*

The translation of *in vitro* data to *in vivo* effects has always been a challenge and it is not a MPS specific problem. However, as we foresee that these advanced cell cultures are better predicter of the *in vivo* processes, the interpretation of the data is crucial. The question is what we should compare the data generated with NAMs to. As the NAMs often are using human cells the species differences to traditional animal models might poses another challenge. In toxicology, the human data is often missing, and we are still extrapolating from the animal despite knowing the animals are poor predictors of the human outcomes (Harrison, 2016; Cavero et al., 2019). For this reason, many pharmaceutical companies have started to request animal based MPS, especially dogs. Animal MPS-derived results can be compared with whole animal responses and would then increase our confidence in the human MPS ability to represent human outcomes. But what if animal MPS perfectly correlates with human MPS response but not with the animal *in vivo* response or both MPS and *in vivo* animal models do not correlate with human MPS? This might be the next challenge to address as more data is being generated. Another challenge with iPSC-derived 3D brain models is how the *in vitro* differentiation process is comparable with human primary cells. Often cells are still immature, when effects are assessed and especially, if neurodegenerative outcomes are investigated, this could be a challenge (reviewed in Doss and Sachinidis, 2019). For example, would aged dopaminergic neurons from human brain behave the same as iPSC-derived ones? The advantage of 3D brain models is that they can be kept in cultures much longer than traditional monolayer cell systems and several groups are reporting on brain organoids differentiated for several months and even a year (Lancaster and Knoblich, 2014; Madhavan et al., 2018; Trujillo et al., 2019). Recently, new methods to senescence cultures further and faster have been explored (Bigagli et al., 2016; Petrini et al., 2017; Burrinha et al., 2019).

## Second Generation Microphysiological Systems

Even though the 3D brain models are complex and more *in vivo* like than the traditional *in vitro* assays, there is still prospects for enhancement. Optimization of 3D brain organoids toward more complex MPS by combining 3D with microfluidics, chips and introducing missing cellular and barrier components will bring those systems to a true MPS—MPS 2.0 (Table 1 and below).

### Immune System

One of the major limitations of most current human *in vitro* models, not only 3D, is the lack of the immune component. It is crucial for DNT to incorporate immune cells (microglia) into the brain organoids as they play a big role in the developing brain and are key for neuroinflammation a crucial tissue response to environmental stress (Werneburg et al., 2017; Wright-Jin and Gutmann, 2019; Badimon et al., 2020). Protocols for iPSC-derived microglia have been developed (Abud et al., 2017; Haenseler et al., 2017) and publications of microglia incorporation into 3D cultures are emerging (Abreu et al., 2018; Ormel et al., 2018; Bodnar et al., 2021; Xu et al., 2021). However, other cells of the immune system might be important for the developing brain as well (Zhou et al., 2021) and we might see more research going in this direction with incorporating of T and B cells to the MPS.

### Barriers

Blood Brain Barrier (BBB) and placental barrier are other essential components to include in neurotoxicity and DNT studies. There are several models of BBB developed: co-cultures of neurons, astrocytes, endothelial cells, including transwell (Lippmann et al., 2014; Faal et al., 2019; Ohshima et al., 2019), spheroid (Cho et al., 2017; Nzou et al., 2018; Nzou et al., 2020) and chip-style (Yeon et al., 2012; Wang YI et al., 2017) systems. There are few *in vitro* models of the placenta barrier (Haider et al., 2018; Turco et al., 2018; Sheridan et al., 2020) but to our knowledge they were never applied in DNT. The placenta protects the fetus from insult, while fetal BBB is not fully developed yet. Thus, any disturbance or loss of functional placenta integrity could contribute to DNT effects. Future research is needed to investigate how xenobiotics can interfere with the interaction of the placenta and the developing fetus, including the brain.

### Cellular Composition

3D brain models have different levels of complexity as compared to the *in vivo* brain. Spheroids are 3D but lack brain architecture while brain organoids have more defined structures such as layering of the cortex (Figure 1). However, all of them lack the physiological relevant cellular composition, i.e., neuron/glia ratio. Thus, all these models need further adjustments to bring them closer to the *in vivo* brain. Modification of the medium, addition of growth factors and signaling molecules, might help to modulate the ratio and promote glia differentiation to bring it to more physiological-relevant distribution of different cell types (neurons/glia—1:1) (reviewed in von Bartheld et al., 2016). Through the implementation of microfluidic systems, growth factor gradients and biomaterials such as extracellular matrix, the special-temporal signaling may become possible (reviewed in Roth et al., 2021). This may further stimulate the maturation and bring histoarchitecture to a more *in vivo* like level (Kilic et al., 2016; Cho et al., 2021).

### Patient Derived iPSC Brain Models

Currently, the different protocols to generate and maintain iPSC may induce variability between different cell lines. Therefore, there can be a challenge to understand if a cell line derived from a specific patient is behaving differently due to the disease or the reprograming and maintenance protocols used. Even if the same protocol is used the different clones from the same patient have shown differences due to reasons we still have limited knowledge about (reviewed in Doss and Sachinidis, 2019; Volpato and Webber, 2020). The use of CRISPR/Cas9 modified isogenic cell lines is one possible solution to overcome this issue (Wang P et al., 2017). However for many diseases, the genetic contribution is more complex than a mutation in one gene or even unknown (idiopathic autism as an example). Another option to increase power of the experiments conducted in patient-derived iPSC lines is to increase number of donors and clones used for experimental set-ups. At least six donors per condition should be considered. As the protocols get more standardized and the reproducibility of 3D brain models enhanced, the use of patient-derived iPSC for neurodevelopmental disorders will likely increase.

### Combination of 3D Brain Models With Other Advanced Technologies (Single Cell Sequencing, High Throughput High Content Imaging, Bioengineering)

Over the last decade there has been tremendous evolution in cell-based techniques. It is foreseen that these will be further developed and be more commonly applied in different fields including neurotoxicology and DNT. Going forward there will likely be more real time non-invasive measurements in brain models, e.g., tracing of individual cells for an extended period of time using live-imaging techniques. Other high-content techniques such as single cell sequencing and omics methods will get cheaper and simpler to use. Single cell sequencing use in combination with brain organoids is increasing, as it allows to track the cell-specific molecular changes as well as lineage composition etc. (Kanton et al., 2019; Kanton et al., 2020; Sawada et al., 2020). Moreover, the culturing techniques to generate 3D structures in a more physiological relevant architecture will be developed. Novel endpoints more linked to human data and diagnosis are expected to be developed, such as biomarker discovery. Once substantial MPS data has been generated, it has the potential to also enhance the establishment of more *in vivo* relevant approaches.

### Organ-Organ Interactions

It is well recognized that organ-organ interactions can influence the toxicity of chemicals, e.g., the metabolic activity of the liver, filtration by the kidney, and the microbiome in the gut. The combination of different organs, including the brain, as in the human-on-chip approach (Novak et al., 2020), will be more broadly used. For the developing brain, not only the other organs within the fetus are important, the interaction with the mother’s physiology including the hormonal system, inflammatory responses, and stress also play a role. The future MPS will likely incorporate many of these factors known to contribute to DNT.

## Conclusion

The 3D brain models recapitulating the human *in vivo* brain are increasingly applied in the fields of neuroscience, neurotoxicology and neurological diseases. However, there are still several improvements to aim for: increased reproducibility and standardization, reduced costs, increased throughput, and assay optimizations. Certainly, those models will continue to enhance, get more physiological relevant with, e.g., incorporation of immune cells, engineered cells, and broadly used patient-derived iPSC. The translation to the human patients will hopefully support the development of new biomarkers, personalized medicine, mechanistic knowledge about neurological disorders, potential treatments and an understanding how xenobiotic exposure contributes to DNT.
